# Molecular epidemiological study of clinical *Acinetobacter baumannii* isolates: phenotype switching of antibiotic resistance

**DOI:** 10.1186/1476-0711-12-21

**Published:** 2013-08-21

**Authors:** Chang-Hua Chen, Chieh-Chen Huang

**Affiliations:** 1 Division of Infectious Diseases, Department of Internal Medicine, Changhua Christian Hospital, No. 135 Nanshiao street, Changhua 500, Taiwan, R.O.C; 2 Department of Nursing, College of Medicine & Nursing, Hung Kuang University, No. 1018, Sec. 6, Taiwan Boulevard, Shalu District, Taichung City 43302, Taiwan, R.O.C; 3 Department of Life Science, College of Life Science, National Chung Hsing University, No. 250 Kuo-Kuang Road, Taichung 402, Taiwan, R.O.C

**Keywords:** *Acinetobacter baumannii*, Pulsed field gel electrophoresis, Multilocus sequence typing, Short tandem repeat, Phenotype switch

## Abstract

**Background:**

The presence of clinical *Acinetobacter baumannii (A. baumannii)* isolates with differing antibiotic resistance phenotypes in the same patient causes difficulties and confusion in treatment. This phenomenon may be caused by reasons such as cross-infection from neighboring patients that switches to different *A. baumannii* strain, natural mutation of *A. baumannii*, inducing of different antibiotic resistance genes expression or acquisition of genes conferring resistance from another source. To elucidate this question, clinical *A. baumannii* strains, isolated from the same individual patients, showed antibiotic resistance phenotypes switching during the same hospitalization period, were attentively collected for further analysis. Molecular approaches for phylogenetic analysis, including pulsed field gel electrophoresis, multilocus sequence typing, and short tandem repeat analysis, were employed for the chronological studies.

**Findings:**

Our results showed that antibiotic resistance phenotype switching could have occurred as a result through both cross-infection and natural mutation roots. Our results also suggest that rapid phenotype switching between paired isolates could occur during one single course of antibiotic treatment.

**Conclusions:**

Though cross infection caused antibiotic resistance phenotype switching does occur, natural mutation of *A. baumannii* isolates is particularly cautious for antibiotic treatment.

## Findings

### Introduction

*Acinetobacter baumannii* (*A. baumannii )* was identified from the environment in the early twentieth century, and has been isolated worldwide. The rapid spread of multidrug-resistant *A. baumannii* (MDRAB) in clinical settings has made choosing an appropriate antibiotic to treat these infections difficult for clinicians. *A. baumannii* within genetically uniform populations exhibit significant phenotypic variability [1]. For example, antibiotic susceptible clinical *A. baumannii* isolates can develop antibiotic resistant phenotypes, in a process called phenotype switching. Such phenotype switching can be perplexing for clinicians, in both interpreting microbiological results and choosing effective antibiotics.

Shanley *et al*. showed that *Acinetobacter calcoaceticus* can naturally uptake, incorporate, and stably maintain DNA *in vitro*[2]. Only a few reports have mentioned the rapid adaptation of *A. baumannii* isolates in a hospital environment [3,4]. Determining whether the multiple resistance phenotype switching is due to cross-infection from neighboring patients or from natural mutation of the same *A. baumannii* isolate is important because of the different strategies needed to resolve the clinical issues. Here we report the rapid change of resistance phenotype of clinical *A. baumannii* isolates from individual patients during the same admission at a single medical institution in Taiwan.

### Material and methods

#### Isolates and phenotyping

We designed a chronological study to collect pairs of phenotypically-identified *A. baumannii* isolates from individual patients during the same hospitalization period at Changhua Christian Hospital (CCH). Pool of samples for further analysis was collected from January 1 1998 to December 31 2008. Among those samples, there were three pairs of clinical *A. baumannii* isolates from CCH that met the inclusion criteria: Pair 1 (isolates 29-4 and 29-43, numbered according to their position in the CCH Bacterial Bank), Pair 2 (isolates 10-18 and 10-10), and Pair 3 (isolates 14-91 and 14-81). Phynotypic method to identify those *A. baumannii* isolates is using a Vitek-2 System (BioMerieux, Marcy l'Etoile, France). And, the isolates were identified according to 16S ribosomal RNA region at the molecular level, as previously described [5].

#### DNA isolation, ribotyping, and detection of short tandem repeats (STR) from clinical *A. baumannii* isolates

Genomic DNA was isolated from three colonies from an overnight culture grown on blood agar plates (bioMérieux, Den Bosch, The Netherlands) using a Bacterial Genomic DNA Isolation Kit III according to the manufacturer’s instructions (Roche, Mannheim, Germany). The ribotype pattern was interpreted to identify the group to which each strain belonged, as previously described [6]. The primer pair *REP1R-I* (5-IIIICGICGICATCIGGC-3) and *REP2-I* (5-ICGICTTATCIGGCCTAC-3) [7] was used to amplify putative *REP*-like elements from the bacterial DNA.

#### Pulsed field gel electrophoresis

We followed a standard protocol for pulsed-field gel electrophoresis (PFGE) analysis of the *A. baumannii* isolates. In brief, *A. baumannii* were plated on blood agar and incubated in a 5% CO_2_ atmosphere at 35°C for 16–24 h. Plug slices were digested with 20 U of *Sgr*AI. The DNA fragments were then separated in 1% Seakem Gold agarose gels (FMC BioProducts) at 14°C using a Bio-Rad CHEF DRIII PFGE system (Bio-Rad Laboratories, Hercules, CA, USA). Gels were run in 0.5× Tris-borate-EDTA (TBE; pH 8) at a 120° fixed angle and a fixed voltage (6 V/cm), with pulse intervals from 4–40 s for 20 h. Following staining and imaging, the chromosomal DNA restriction patterns produced by PFGE were interpreted using Tenover’s categorization [8].

#### Multilocus sequence typing

Multilocus sequence typing (MLST) was performed according to the method of Bartual *et al*. [9]. In brief, housekeeping genes for MLST were selected based on their sequence availability in GenBank, on prior studies of the phylogenetic relationships for the genus *Acinetobacter*, and on their use in MLST schemes for other bacterial species [1,10-12]. PCR primers were chosen from previous studies or were newly designed for amplification of the seven selected genes: citrate synthase (*gltA*), DNA gyrase subunit B (*gyrB*), glucose dehydrogenase B (*gdhB*), homologous recombination factor (*recA*), 60 kDa chaperonin (*cpn60*), glucose-6-phosphate isomerase (*gpi*), RNA polymerase 70 factor (*rpoD*). All PCR amplifications were performed in a MasterCycler gradient instrument (Eppendorf, Hamburg, Germany). Sequencing of internal fragments (~450 bp in size) of the selected housekeeping genes was performed in an ABI Prism 377 sequencer using the ABI Prism BigDye terminator cycle sequencing ready reaction kit v. 2 (Applied Biosystems, Foster City, CA, USA) according to the manufacturer’s recommendations.

### Results

**Figure 1:**
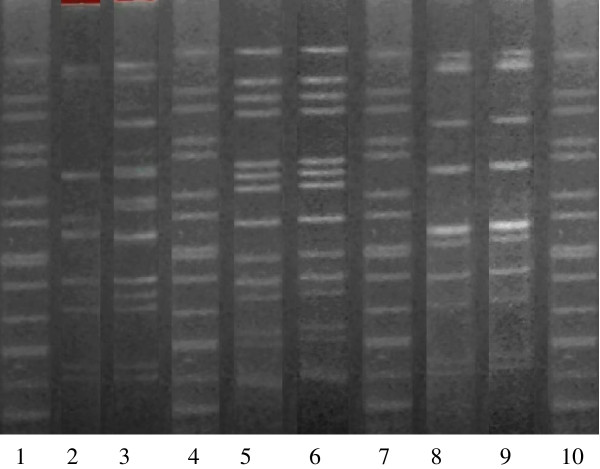
**PFGE fingerprints of three pairs of clinical *A. baumannii *isolates following digestion with the *Sgr*AI restriction enzyme.**

We collected clinical and microbiological profiles focusing on the three pairs of *A. baumannii* isolates from patients during an individual hospitalization. All three patients stayed at our institute for at least two weeks, and all of them received antibiotics following identification of the *A. baumannii* isolates (Table 1). The antibiotic susceptibility of clinical *A. baumannii* isolates are listed in Table 2. Four PFGE fingerprint patterns were detected in the three pairs of *A. baumannii* isolates in Figure 1. Furthermore, there appears to be a clear link of cross-infection between the PFGE types and the clinical data available for the isolates. Interpretation of the MLST data revealed that more than half of the MLST allelic profiles from the three pairs of *A. baumannii* isolates differed from those already in *A. baumannii* MLST databases (http://pubmlst.org/abaumannii/)[13]. Comparison of the sequence types (ST) of the three paired *A. baumannii* isolates showed similarity between the 29-4 and 29-43 *A. baumannii* isolates, especially in the allelic profiles of *gltA, gdhB, recA,* and *rpoD* (Tables 3, 4). However, there was a difference between the 14-91 and 14-81 paired isolates, especially in the allelic profiles of *recA, cpn60*, and *rpoD* (Tables 3, 4). These results indicated that isolates 29-4 and 29-43 are the same isolate, and that both paired 14-91 and 14-81 isolates and paired 10-18 and 10-10 isoaltes are different isolates (Tables 3, 4). The fingerprint patterns of the STRs were quite varied (Tables 3, 4). It is particularly interesting that rapid phenotype switching between the paired isolates (29-4 and 29-43) could occur during one course of antibiotic treatment.

**Table 1:** The time line of antimicrobial agents prescription

**Event**	**Date**	**Patient number one, Pair 1**	**Date**	**Patient number two, Pair 2**	**Date**	**Patient number three, Pair 3**
Admission day	July 29	Admission day	July 2	Admission day	June 3	Admission day
Antimicrobial agent	July 29 and Aug 4	Cefuroxime	July 2 and July 19	Ampicillin-sulbactam	June 3 and June 10	Ampicillin-sulbactam
Isolation day	Aug 2	Isolates 10-10 from sputum	July 19	Isolates 29-43 from sputum	June 7	Isolates 14-91 from sputum
Antimicrobial agent	Aug 4 and Aug 7	Ceftazidime	July 19 and July 26	Piperacillin-tazobactam	June 10 and June 17	Cefotaxime
Antimicrobial agent	Aug 7 and Aug 10	Piperacillin-tazobactam	July 26 and Aug 1	Levofloxacin	June 17 and June 24	Piperacillin-tazobactam
Antimicrobial agent	Since Aug 10	Imipenem-cilastatin	Since Aug 1	Imipenem-cilastatin	Since June 24	Meropenem
Isolation day	Aug 16	Isolates 10-10 from abscess	Aug 18	Isolates 29-4 from tip of central catheter	July 7	Isolates 14-81 from sputum

**Table 2:** **Antibiotic susceptibility of clinical *Acinetobacter baumannii *isolates**

**Number of isolate**	**10-10**	**10-18**	**29-43**	**29-4**	**14-91**	**14-81**
Date of isolation	2-Aug	16-Aug	19-Jul	18-Aug	9-Jun	7-Jul
Time of isolation	PM 02:38:55	PM 03:10:10	PM 04:21:03	PM 02:47:06	AM 09:12:47	AM 10:18:53
Diagnosis	pneumonia	Soft tissue infection	pneumonia	Catheter-related infection	pneumonia	pneumonia
Specimens	Sputum, tracheal aspirate (suction)	abscess	Sputum, tracheal aspirate (suction)	Tip of central catheter	Sputum, tracheal aspirate (suction)	Sputum, tracheal aspirate (suction)
Antibiotic	Minimum inhibitory	concentrations	(ug/mL)			
AN	8	128	8	128	8	64
SAM	32	128	32	128	32	128
CTZ	8	64	8	64	8	32
LVF	2	128	2	128	2	64
IMP	2	16	2	16	2	16
PIP-TAZ	8	256	8	256	8	256
CRO	8	128	8	128	8	64
CFP	8	256	8	256	8	128
MEP	4	32	4	32	4	16

Notes: The susceptibility tests were performed using the Vitek-2 GN card (Biomerieux, Marcy l'Etoile, France). The results were interpreted using the CLSI breakpoints (Clinical and Laboratory Standards Institute Performance Standards for Antimicrobial Susceptibility testing; Twenty-First Information Supplement. CLSI document M100-S21, CLSI, Wayne, PA; 2011).

AN: amikacin; SAM: ampicillin-sulbactam; CTZ: ceftazidime; LVF: levofloxacin; IMP: imipenem-cilastatin; PIP-TAZ: piperacillin-tazobactam; CRO: ceftriaxone; CFP:cefepime; MEP: meropenem; colistin and tigecycline and polymyxin B were not provided by Vitek-2 System.

**Table 3:** **The results of pulsed field gel electrophoresis, multilocus sequence typing, and short tandem repeat analysis of three pairs of *A. baumannii *isolates**

**Isolates**	**Geno type(†)**	**PFGE type**	**ST Type(‡)**	***gltA***	***gyrB***	***gdhB***	***recA***	***cpn60***	***gpi***	***rpoD***	**STR type**
10-18	Ab 1	A	this study (a1)	6	this study	this study	this study	this study	this study	this study	I
10-10	Ab 2	A	this study (a2)	11	this study	this study	this study	this study	this study	this study	II
29-4	Ab 3	B	this study (a3)	1	this study	3	2	2	this study	3	III
29-43	Ab 3	B	this study (a4)	1	3	3	2	this study	7	3	IX
14-91	Ab 4	C	this study (a5)	1	this study	this study	2	1	23	18	X
14-81	Ab 5	D	this study (a6)	1	this study	3	2	2	this study	3	XI

Notes

Sequences of amplified genes were compared with sequences from the *A. baumannii* MLST website (http://pubmlst.org/abaumannii/).

ST: sequence type.

† the given name of the genotype is defined as *A. baumannii +* number (Ab + number).

‡ the given name of the genotype is defined as *Acinetobacter* isolate + number (a + number).

**Table 4:** **Aligmnent for three pairs of *Acinetobacter baumannii *siolates**

**10-10 VS 10-18**	**gltA**	**gyrB**	**gdhB**	**recA**	**cpn60**	**gpi**	**rpoD**
Length	484	927	744	371	454	358	860
Score	684	1218	1258	667	819	662	1589
Identities	446/484	835/922	699/707	361/361	446/447	358/358	860/860
Difference	38	87	8	0	1	0	0
Gaps	0/484	4/922	4/707	0/361	1/447	0/358	0/860
29-4 VS 29-43	gltA	gyrB	gdhB	recA	cpn60	gpi	rpoD
Length	484	936	396	371	454	363	513
Score	894	806	732	686	778	464	948
Identities	484/484	450/457	396/396	371/371	421/421	265/272	513/513
Difference	0	7	0	0	0	7	0
Gaps	0	0	0	0	0	0	0
14-91 VS 14-81	gltA	gyrB	gdhB	recA	cpn60	gpi	rpoD
Length	484	932	396	371	421	360	513
Score	894	1701	399	686	773	392	931
Identities	484/484	924/925	318/369	371/371	420/421	253/273	510/513
Difference	0	1	51	0	1	20	3
Gaps	0	1	0	0	0	2	0

### Discussion

The is the first report of phenotype switching of antibiotic resistance in clinical *A. baumannii* isolates in individual patients during the same hospitalization in Taiwan. While *A. baumannii* has been reported previously in Taiwan, and prolonged administration of broad-spectrum antibiotics will induce the development of antibiotic resistance in clinical *A. baumannii* isolates, little is known about the current clinical situation. It was demonstrated that a important evolutionary change of a single genotype was fundamental to the continuous rise observed in the number of *A. baumannii* infections [4].

The current study suggests that natural transformation and mutation of genotypes occurred in clinical *A. baumannii* isolates 29-43 and 29-4 on the basis of PFGE. We used three methods to determine the genetic similarity of the paired *A. baumannii* isolates: PFGE, MLST, and STR. Snelling *et al*. described a PCR assay using repetitive extragenic palindromic sequences to type *A. calcoaceticus* and *A. baumannii* strains [14], while Alcala *et al*. characterized a meningococcal epidemic wave using a MLST method [15], similar to that used in our study. The congruence between the MLST, PFGE, and STR data suggests that the findings of the current study are sound; however, further experiments are required to prove the relationships among the paired isolates.

In this study, we discovered natural mutation and rapid change of antibiotic resistance phenotype of clinical *A. baumannii* isolates from an individual patient. This is alarming as this particular clone seems to be able to effectively fill niches that were essentially uninhabited by *A. baumannii* in the past. Even in a relatively closed environment, the isolates of identical PFGE fingerprint patterns showed a variety of MLST patterns. Apparently, the MLST patterns of paired isolates 29-4 and 29-43 are capable of withstanding background mutation. It is possible that the mutation rate of this particular isolate may contribute to its success in coping with different environments.

### Conclusions

This study provides novel insight into the clinical problem of whether different *A. baumannii* isolates from the same patient are due to cross-infection from neighboring patients or from natural mutation. This is important for clinicians because the treatments for the two causes are different. The approach for the first phenomenon is to enhance contact precautions in the clinical practice, whereas the second is the stepwise prescription of different antibiotics.

## Availability of supporting data

None.

### Ethical approval

Not required.

## Abbreviations

A. baumannii: *Acinetobacter baumannii*; CCH: Changhua christian hospital; cpn60: 60 kDa chaperonin; gdhB: Glucose dehydrogenase B; gltA: Citrate synthase; gpi: Glucose-6-phosphate isomerase; gyrB: DNA gyrase subunit B; MDRAB: Multidrug-resistant *Acinetobacter baumannii*; MLST: Multilocus sequence typing; PFGE: pulsed-field gel electrophoresis; recA: Homologous recombination factor; rpoD: RNA polymerase 70 factor; ST: Sequence types; STR: Short tandem repeats.

## Competing interests

Both authors declare that they have no competing interests.

## Authors’ contributions

CHC and CCH designed and performed this study. CCH analyzed the data regarding the infectious diseases and wrote the manuscript. Both authors read and approved the final manuscript.
